# Mothers say “baby” and their newborns do not choose to listen: a behavioral preference study to compare with ERP results

**DOI:** 10.3389/fnhum.2015.00153

**Published:** 2015-03-25

**Authors:** Christine Moon, Randall C. Zernzach, Patricia K. Kuhl

**Affiliations:** ^1^Department of Psychology, Pacific Lutheran UniversityTacoma, WA, USA; ^2^Wilford Hall Ambulatory Surgical Center, 59th MDWJBSA-Lackland, TX, USA; ^3^Institute for Learning and Brain Sciences, University of WashingtonSeattle, WA, USA

**Keywords:** auditory, behavior, fetal, learning, motivation, neonatal, non-nutritive sucking, preference

## Abstract

Previously published results from neonatal brain evoked response potential (ERP) experiments revealed different brain responses to the single word “baby” depending on whether it was recorded by the mother or an unfamiliar female. These results are consistent with behavioral preference studies in which infants altered pacifier sucking to contingently activate recordings of the maternal vs. an unfamiliar female voice, but the speech samples were much longer and information-rich than in the ERP studies. Both types of neonatal voice recognition studies imply postnatal retention of prenatal learning. The preference studies require infant motor and motivation systems to mount a response in addition to voice recognition. The current contingent sucking preference study was designed to test neonatal motivation to alter behavior when the reward is the single word “baby” recorded by the mother or an unfamiliar speaker. Results showed an absent or weak contingent sucking response to the brief maternal voice sample, and they demonstrate the complementary value of electrophysiological and behavioral studies for very early development. Neonates can apparently recognize the maternal voice in brief recorded sample (previous ERP results) but they are not sufficiently motivated by it to alter sucking behavior.

Recent electrophysiological and brain imaging studies have advanced our understanding of the development of very early perception and learning through measurement of brain activity in response to specific events (May et al., 2011; Partanen et al., 2013; Kuhl et al., 2014). When there are differential responses to familiar vs. novel stimuli, learning and memory can be inferred. These methods permit localization to specific brain areas, and they are valuable for mapping the immature brain as it undergoes rapid development. It is, of course, ideal when brain activity can be linked to behavior, and the perception-action circuit is illuminated. Documenting the link can be difficult in early development because the competence-performance distinction (Chomsky, 1965) is particularly relevant. If the brain shows that a stimulus is recognized, there may be no corresponding measurable behavior because the individual cannot yet execute a motor response. A second consideration is that the path to behavior includes not only motor competence but also motivation. The newborn brain may detect an event, recognize it, and may be capable of rendering a motor response, but if the event is not sufficiently motivating, the infant will not mount the response.

Results of several experiments show that the newborn brain responds differentially to even a brief snippet of the maternal vs. a stranger female voice (Deregnier et al., 2000; deRegnier et al., 2002; Siddappa et al., 2004; Therien et al., 2004). In several studies, scalp recordings of brain activity in sleeping newborns using evoked response potentials (ERPs) used the brain’s mismatch detection response to show this discrimination. This procedure requires that the stimuli be brief so the brain’s rapid response can be time-locked to stimulus presentation. In the newborn ERP studies, the mother and stranger voice recordings were each limited to a brief sample of the word “baby” that averaged 750 ms. The midline brain response to both the maternal and novel voices was a positive wave appearing at 290 ms and that has been interpreted as reflecting auditory stimulus detection of both types of stimuli. For the novel voices only, the positive wave was accompanied by a negative slow wave that was interpreted as a response to novelty (deRegnier, 2005). The lack of a similar response to the maternal voice implies recognition of a familiar sound. The recognition response has been detected in newborns from uncomplicated pregnancies and births, whereas in newborns of iron-deficient (Siddappa et al., 2004) mothers and in extremely premature newborns (Therien et al., 2004), the differences are attenuated or absent. These ERP differences were interpreted as showing compromised recognition memory development in the infants of complicated pregnancies or deliveries.

Prior to these ERP results, it was not known whether newborns could even recognize their mothers’ voices without the prosodic information present in lengthy and acoustically rich samples of talking or reading. Doubt about the limits of newborn voice recognition arose from analysis of intrauterine voice recordings (Querleu et al., 1988) and from behavioral experiments using only lengthy and acoustically rich voice samples. For a review, see Moon and Fifer (2000). It is now apparent that brief and relatively uninformative voice samples are sufficient for a differential brain response.

There have been several behavior-based investigations of maternal voice recognition. DeCasper and Fifer showed that neonates alter their behavior to selectively activate the sound of the mother reading a nursery rhyme (DeCasper and Fifer, 1980). Because the results were based on infants who had had no more than 12 h postnatal experience with their mother, their selective response to the maternal voice implied postnatal retention of prenatal learning. In the experiment, sound presentations of the maternal voice were contingent on infants altering their sucks on a pacifier. The authors compared the observed behavior to that of non-human infants who express “perceptual preference” and “proximity-seeking behavior” p.1176 (DeCasper and Fifer, 1980). Developmental psychobiologists and cognitive development researchers have described this kind of behavior as a “listening bias” (Vouloumanos and Werker, 2004), “operant choice” (Granier-Deferre et al., 2011), “operant learning” (Floccia et al., 1997) and “operant-choice preference” (Aldridge et al., 2001). The latter terms presuppose differential reinforcing value of the stimuli and motivation to respond. A contingent sound-sucking experiment using yoked-control methodology showed that the contingency is important in increasing sucking rates (Floccia et al., 1997). Thus, a measurable difference in neonatal preference or choice offers the opportunity to study motivation at the beginning of life.

Since 1980, experiments using contingent pacifier sucking have replicated and extended two main ideas: (i) humans learn before birth; and (ii) prenatal learning can affect postnatal behavior. The maternal voice preference has been robust in demonstrating learning in the womb. It has held whether the voice samples were highly melodic and rhythmic recordings of mothers reading nursery rhymes (DeCasper and Fifer, 1980) or relatively monotone adult conversation (Fifer and Moon, 1989). Although neonates respond to a simple maternal voice recording, they prefer a version that has been low pass filtered to simulate the voice *in utero* (Fifer and Moon, 1995; Spence and Freeman, 1996). A newborn experiment with fathers’ and comparison male voices failed to show a preference, despite newborn discrimination of the two voices (DeCasper and Prescott, 1984). During the neonatal period, infants have expressed preferences for other sounds available *in utero* such as the mother’s native vs. a foreign language (Mehler et al., 1988; Moon et al., 1993). The native over foreign language preference was replicated and extended in a study showing that, for infants of bilingual mothers, her two languages receive equivalent responses (Byers-Heinlein et al., 2010). Perhaps the most convincing example of prenatal learning’s effect on postnatal preference are the results of an exposure study in which mothers read a nursery rhyme out loud during pregnancy. Newborns sucked more to activate the familiar rhyme vs. a novel rhyme, regardless of whether the voice was maternal (DeCasper and Spence, 1986). Thus, there is converging evidence from 35 years of laboratory research that shortly after birth infants are capable of employing prenatal experience, motor control, and the motivational system to mount a behavioral response when the consequence is hearing a familiar sound.

What has not been known up to now is whether the brief and recognizable sample of the maternal voice saying a single word is sufficiently motivating for newborns to mount a preference response. What follows is a description of an experiment using the preference procedure in which the consequence of infant sucking on a pacifier was the delivery through headphones of brief maternal and non-maternal voice samples of the word “baby”. Thirty-six infants were in the experimental group of Mother-Stranger (MS) infants for whom the stimuli were the maternal vs. a stranger female voice. Twenty-four infants were in the Stranger-Stranger (SS) control group who heard two different non-maternal female voices. If the brief snippets of mother’s voice are sufficiently motivating for newborns to alter sucking behavior to show a preference, then (Kuhl et al., 2014) sucking during opportunities to produce the maternal voice should exceed those for the stranger voice, and (May et al., 2011) sucking by the MS group should exceed that of the SS group.

## Materials and Methods

### Participants

Sixty term neonates (*M* age = 31 h, SD = 10.3) each completed an experimental session prior to discharge from the mother-baby postpartum unit of a military medical center. They had no documented antenatal or birth complications, no risk factors for hearing loss (American Academy of Pediatrics Joint Committee on Infant Hearing, 2007), and English was the primary language spoken in the home. Infants were assigned to one of two conditions. In the MS Condition (*N* = 36), the stimuli were mother’s and a stranger female’s voice. In the Stranger-Stranger Condition (*N* = 24), the stimuli were two voices of unfamiliar females. In order to be included in the analysis, infants were required to complete a 10 min session with no more than two consecutive or three non-consecutive minutes in which no sucking occurred. Study sessions were terminated immediately if the infant became excessively fussy or cried. The data from 29 infants were excluded from analysis due to drowsiness (*n* = 18), crying/fussiness (*n* = 3), inconsistent sucking not apparently due to arousal state (*n* = 7), or experimenter error/equipment problems (*n* = 1).

### Apparatus and Stimuli

Infants sucked on a Gerber Little Suzy Zoo pacifier fitted with a plastic tube connected to a Becton-Dickinson P23 × L pressure transducer that provided input to a Grass Telefactor CP122 Strain Gage Amplifier. The analogue output of the amplifier was converted to a digital signal by a Data Translation DT2814 data acquisition board that was connected to a Gateway 486 PC equipped with a ProAudio 16 sound card. Custom software recorded sucking pressure, controlled stimulus delivery, and created a summary data file. Analog pressure changes were converted into digital signals and served as input for a Gateway 486 PC. The computer delivered stimuli to the infants via Grado SR225 earphones suspended from a custom-made adjustable plexiglass frame that fit into the infant’s bassinet. Voice stimuli were recorded using an Electro-Voice PL88H Microphone on a laptop computer. Analysis of the recorded speech samples was conducted using Signalyze Speech and Sound Analysis software on an Apple computer.

Each of 53 stimuli consisted of one naturalistic token of the word “baby” spoken in a woman’s voice. Talkers had been instructed to say the declarative sentence, “He’s/she’s a baby” with a falling intonation contour. The statement was followed by four repetitions of the word “baby” using the same intonation contour. One voice token for each speaker was chosen on the basis of absence of background sound, clarity, loudness and presence of falling intonation contour. The mean duration of voice stimuli was 480.0 ms (s.d. 92.3). For the MS group, the Mother and Stranger stimuli did not significantly differ in duration, nor did the two voices in the SS condition. Pairs of stimuli were matched for loudness by two adult listeners and were presented at about 66 dBA as measured by a sound level meter (Bruel and Kjaer Model 2235) placed midway between the headphones.

### Design and Procedure

The study was conducted according to a protocol that was approved by the medical center and university (PLU) ethics boards. For the MS infants, the stimuli were available during five one-minute periods of stimulus voice alternations for a total of 10 min (Cowan et al., 1982; Sansavini et al., 1997; Vouloumanos and Werker, 2007). The MS stimuli were counterbalanced for order of presentation (Mother First, *N* = 17, Stranger First, *N* = 19) and were delivered on a partial reinforcement schedule of a minimum of two qualifying sucks in quick succession for reinforcement. The Stranger-Stranger infants (SS) heard two different unfamiliar female voices, the availability of which alternated in one-minute intervals for 10 min. Each voice was thus available for five one-minute periods.

Parental informed consent was obtained in the mother-infant hospital room according to university- and medical center-approved protocols. For infants in the MS group, a recording of maternal voice was obtained at the bedside for subsequent editing. Each mother received a walkie-talkie to call when her infant appeared to be in a quiet and alert state. Infants were transported to the study room in the mother-baby unit where the headphone frame was placed in the bassinette and headphones were fitted next to the infants’ ears. Prior to the beginning of the session, an experimenter swaddled the infant either snugly or loosely, based on the best judgment about supporting a quiet and alert state for 10 min. The experimental pacifier was given to infants and was held in place by an experimenter who listened to masking music through headphones during the session. After subjects demonstrated consistent sucking on the pacifier, usually no more than two to three minutes, the study session began by recording 1 min of baseline sucking data at the conclusion of which a threshold was established for stimulus delivery. The threshold was set at the 30th percentile of sucking amplitudes produced during the baseline minute. That is, sucks that were above the lowest 30% of baseline suck amplitudes resulted in stimulus activation. This is consistent with previous neonatal contingent sucking studies in which amplitude threshold has ranged from the 20th (Floccia et al., 2000; Vouloumanos and Werker, 2007) to the 50th (Floccia et al., 1997) percentiles of baseline.

## Results

The dependent measure was the number of qualifying (above threshold) sucks per minute. Analyses included the factor of time (sequential minutes of the session) because previous studies have shown the emergence of a preference response over time (Sansavini et al., 1997; Vouloumanos and Werker, 2004, 2007). A D’Agostino-Pearson test of normality was conducted on the mean number of sucks per minute for the sample of 60 newborns, and results were consistent with a normal distribution, *K*^2^ = 2.21, *p* > 0.05. A preliminary independent samples *t*-test was conducted on sucks during baseline for stimulus conditions MS vs. SS. There was a significant difference with greater mean sucks per minute of baseline for the MS group, *M* = 43.0, SD = 17.6 compared to the SS group, *M* = 31.9, SD = 19.2, *t*_(58)_* =* 2.3, *p* = 0.025. Baseline sucks were entered as a covariate in subsequent analyses.

**Figure 1:**
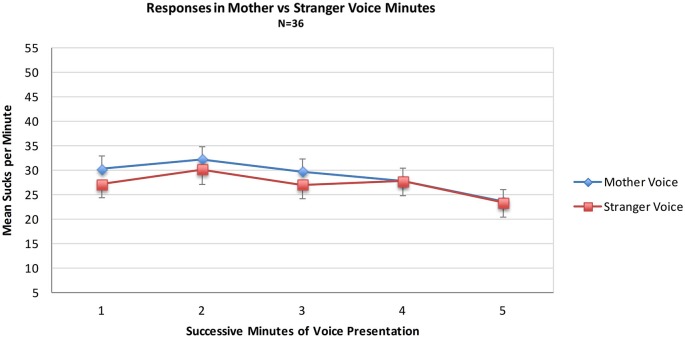
**Mean sucks per minute to the maternal vs. the stranger female voice**. Voice stimuli were contingent on sucking, and the maternal and stranger voices alternated in five one-minute intervals of a 10 min presentation period.

Data from the MS group were used to test the hypothesis that sucks during minutes of opportunity to hear mother’s voice would exceed those of stranger voice minutes. For the MS group (*N* = 36) a mixed 2 (**Voice**) × 2 (**Order** of voice presentation) × 5 (**Minutes**) ANOVA was conducted. There were no statistically significant main effects of the within-subjects variable Voice (Mother Voice sucks per minute: Mean = 28.7, SD = 16.3, Stranger Voice Mean = 27.0, SD = 14.4 or the between-subjects variable of Order. There was no main effect of Minutes nor were there any significant interaction effects. See Figure 1; Table 1.

**Table 1:** **Analysis of variance results for the Mother-Stranger group (*N* = 36)**.

Source	*Df*	*F*	*η*^2^	*p*
Voice	1,33	0.36	0.01	0.55
Minutes	4,132	0.59	0.02	0.67
Order	1,33	2.63	0.07	0.11
Voice × Minutes	4,132	0.49	0.01	0.75
Voice × Order	1,33	2.28	0.07	0.14
Mins × Order	4,132	1.32	0.04	0.27
Voice × Min × Ord	4,132	0.93	0.03	0.45

**Figure 2:**
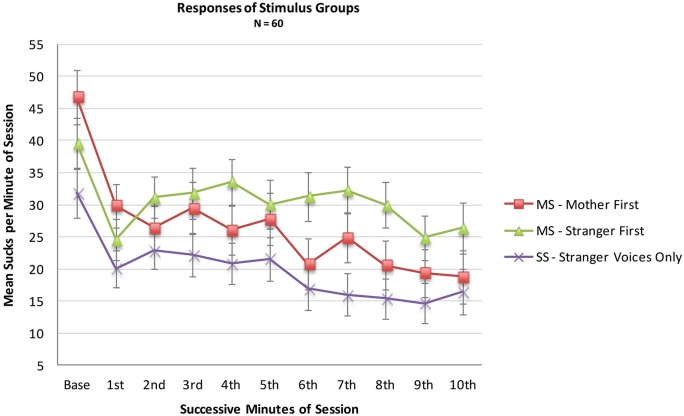
**Mean sucks per minute in the 10 min presentation period of the two MS groups (*N* = 36 total) and the Stranger-Stranger Group (*N* = 24)**. For the MS-Mother first group, the maternal voice was presented in minutes 1, 3, 5, 7, and 9. For the MS-Stranger first group, the maternal voice was presented in minutes 2, 4, 6, 8, and 10.

An analysis was conducted with the entire sample comparing the MS (*N* = 36) and SS (*N* = 24) groups to test whether infant opportunities to suck to activate the maternal voice would result in more sucking overall during the 10 min session, whether or not a higher sucking frequency was confined to the maternal voice periods. A mixed two factor ANOVA included Stimulus Group (2) × Minutes (10). There was no main effect of Stimulus Group (*F*_(1,57)_ = 1.9, *p* = 0.18, *η_p_*^2^ = 0.03), no main effect of Minutes (*F*_(1,513)_ = 1.33, *p* = 0.22, *η_p_*^2^ = 0.02) and no interaction effect of Group X Minutes (*F*_(9,513)_ = 0.89, *p* = 0.22, *η_p_*^2^ = 0.12). See Figure 2.

## Discussion

Neither the direct comparison of responses to the maternal vs. stranger voices nor the indirect comparison in present vs. absent maternal voice provides statistically significant evidence for a preference for the maternal voice. Visual inspection of the pattern of the two MS sub-groups in Figure 2 provides some evidence for a preference response in the transitions mother-to-stranger (Mother First group) and stranger-to-mother (Stranger First group) over 10 min.

One possible explanation for the absent or weak behavioral response to mother’s voice that can be ruled out is that infants could not recognize the maternal voice in the brief sample. Previously published ERP studies demonstrate otherwise, at least in infants without complicated prenatal and birth histories. Moreover, previous sucking experiments have shown that neonates can respond differentially to brief speech samples such as syllables or vowels (Moon and Fifer, 1990; Moon et al., 1992, 1993; Floccia et al., 2000).

Although the newborns were able to discriminate the maternal and the stranger voices, the brief, repetitive sample of the maternal voice was apparently not sufficiently motivating for them to suck to activate it significantly more frequently than the alternative. There are other published reports of newborn failure to show a voice preference in contingent sucking procedures. For example, infants did not differentially suck to activate a recording of father’s vs. a stranger male voice although they could discriminate the voices (DeCasper and Prescott, 1984). Newborns did not show a maternal voice preference when mother’s and stranger voices were whispered (Spence and Freeman, 1996) and they showed no preference when hearing recordings of their bilingual mothers’ two languages (Byers-Heinlein et al., 2010).

Collecting behavioral data from neonates has limitations. There are many sources of variability that are difficult to control such as rapid changes in infant state, competing infant behaviors, and internal perceptual events that are often unspecifiable. In the current study, the attrition rate was 33 per cent. Although this rate is typical or even low for contingent sucking experiments (Floccia et al., 1997, 2000; Vouloumanos and Werker, 2007), it is an indicator of the inherent variability in behavior-based data collection with neonates. Although the current study was conducted by experienced neonatal researchers using a standard contingent sucking protocol, it certainly merits replication, at best with extension to other forms of familiar and unfamiliar stimuli, especially those of caregivers over time as newborns adjust to postnatal life.

Taken all together, the current results add to the literature on neonatal behavioral preferences for sounds, and they complement previously published, electrophysiological results. They inform us about incipient capacities in very early development. In the absence of robust behavioral evidence for discrimination and therefore recognition of mother’s voice, the presence of differential ERP responses confirms that the two auditory signals are, in fact, processed differently by the neonatal brain, at least for infants with uncomplicated pre- and early postnatal histories. Mother’s voice is recognized, even in the impoverished form. The presence or, in this case, the absence of overt behavior informs about the relative salience of the signal and, perhaps, its hedonic valence, something that electrophysiological measures do not provide at present. Mother’s familiar talking or reading voice with its unique and characteristic changes over time in pitch, rhythm and loudness is sufficiently salient and positively valenced that neonates are motivated to act to produce more of it (DeCasper and Fifer, 1980; Fifer and Moon, 1989). Without these characteristics in the sound of the maternal voice, they apparently are not. As newborn brain imaging techniques advance, more will be known about the development of brain organization for perception, memory, motivation and motor control. It will be important to complement this understanding with increasing knowledge about the actions that result.

## Conflict of Interest Statement

The authors declare that the research was conducted in the absence of any commercial or financial relationships that could be construed as a potential conflict of interest.
